# Evaluation of the Memory Enhancing Activity of Dichloromethane Fraction of the Methanol Extract of *Pycnanthus angolensis* Stem Bark on Experimental Models of Memory Impairment

**DOI:** 10.1002/alz.088192

**Published:** 2025-01-09

**Authors:** Patrici Eziafa Chinwuba, Bakre Adewale Ganiyu

**Affiliations:** ^1^ ABUAD, Ado Ekiti, Ekiti Nigeria; ^2^ University of Ibadan, Ibadan Nigeria

## Abstract

**Background:**

*Pycnanthus angolensis* (Welw) Warb., Myristicaceae, is used extensively in ethnomedicine. Numerous health benefits have being ascribed to the use of different parts of *P. angolensis* including its role in cognition enhancement and inflammation. Hence, this study was undertaken to investigate the stem bark of the plant for memory enhancing activity in mice.

**Method:**

The plant material was grinded into powder and extracted by maceration with 80% methanol at room temperature for 48h. This was subsequently fractionated using N‐hexane, Dchloromethane (DCM), and Ethyl acetate. The Dichloromethane (DCM) fraction‐the most potent fraction‐ (25, 50, and 100 mg/kg) was evaluated for memory enhancing activity using the Y maze, Morris Water Maze (MWM) and the Elevated plus maze (EPM), on the D‐galactose plus scopolamine and ketamine induced amnesia model. The antioxidant markers and acetylcholinesterase inhibiting effect of DCM were also evaluated

**Result:**

The results obtained from the behavioural studies indicate that the DCM fraction significantly (p<0.05) increased the alternation behaviour of the mice in the Y maze, decreased the escape latency in the MWM paradigm and decreased the transfer latency (TL) in the EPM. Biochemically, DCM increased Glutathione (GSH), and superoxide dismutase (SOD), but decreased Malondialdehyde (MDA) and acetylcholinesterase (AChE) activity in the brain.

**Conclusion:**

The study therefore suggested that the DCM may possess significant memory enhancing activity, which may be due to the reverser of the activity of scopolamine, thereby, enhancing cholinergic transmission. The attenuation of the effect of ketamine by the DCM may possibly result from an increase in NMDA receptor activity.

